# Design and Functional Characterization of a Novel Abscisic Acid Analog

**DOI:** 10.1038/srep43863

**Published:** 2017-03-08

**Authors:** Xiaoqiang Han, Lun Jiang, Chuanliang Che, Chuan Wan, Huizhe Lu, Yumei Xiao, Yanjun Xu, Zhongzhou Chen, Zhaohai Qin

**Affiliations:** 1 College of Science, China Agricultural University, Beijing, 100193, China; 2 College of Agricultural, Shihezi University, Shihezi, 832000, China; 3 College of Biological Sciences, China Agricultural University, Beijing, 100193, China; 4 Beijing Advanced Innovation Center for Food Nutrition and Human Health, China Agricultural University, Beijing, 100193, China

## Abstract

The phytohormone abscisic acid (ABA) plays a crucial role in mediating plant growth and development by recruiting genetically redundant ABA receptors. To overcome its oxidation inactivation, we developed a novel ABA analog named 2′,3′-benzo-*iso*-ABA (*iso*-PhABA) and studied its function and structural characterization with *A. thaliana* ABA receptors. The (+)-*iso*-PhABA form showed much higher ABA-like activities than (+)-ABA including inhibitory effects on the seed germination of lettuce and *A. thaliana*, wheat embryo germination and rice seedling elongation. The PP2C (protein phosphatases 2C) activity assay showed that (+)-*iso*-PhABA acted as a potent and selective ABA receptor agonist, which is preferred to PYL10. In some cases, (−)-*iso*-PhABA showed moderate to high activity for the PYL protein inhibiting PP2C activity, suggesting different mechanisms of action of *iso*-PhABA and ABA. The complex crystal structure of *iso*-PhABA with PYL10 was determined and elucidated successfully, revealing that (+)-*iso*-PhABA was better coordinated in the same binding pocket compared to (+)-ABA. Moreover, the detailed interaction network of *iso*-PhABA/PYL10 was disclosed and involves hydrogen bonds and multiple hydrophobic interactions that provide a robust framework for the design of novel ABA receptor agonists/antagonists.

Numerous commercial bioactive products, such as synthetic drugs and pesticides, are derived from natural compounds. Unfortunately, natural compounds generally have some drawbacks, including less effectiveness, poor stability and overly complicated structures. It is thus necessary to rationally modify their structure. The plant hormone abscisic acid ((+)-ABA, **1**) has received much attention in recent decades. As a key phytohormone, ABA plays a vital role in regulating many important physiological processes, such as stomatal movements, seed maturation, dormancy, and response to abiotic stresses (e.g., drought, cold, and salinity), as well as induction of α-amylase by gibberellins A_3_ and the expression of proteinase inhibitor II^1^,^2^,^3^,^4^. Based on these vital physiological functions, it also has potential value in agricultural applications. However, the notable shortcomings of ABA, mainly rapidly metabolism and light-induced isomerization, render ABA inactive both *in vivo* and *in vitro* and have considerably limited its agricultural use. Thus, structural modification of natural ABA is an inevitable choice. The development of ABA analogs with simplified structure and high stability accompanying excellent activity remains a challenge for chemists.

Efforts to inhibit metabolic inactivation by ABA 8′-hydroxylase, a P450 monooxygenase that oxidizes ABA at the 8′-carbon atom, generally employone of the following routes: using competitive inhibitors of ABA 8′-hydroxylase^5^ or introducing various functional groups at the 8′-position of ABA, such as trifluoromethyl^6^, vinyl^7^,^8^, methoxymethyl^9^ and ethynyl^10^, to generate ABA analogs that avoid 8′-methyl transformation. However, these compounds are usually difficult to prepare and are thus not yet suited for practical use. The third method is to inhibit the Michael addition reaction of ABA to generate the less active compound of phaseic acid, rather than targeting the 8′-methyl transformation reaction. Preventing the Michael addition earlier in the biosynthesis is accomplished by elevating the electronic density of C2′ by introducing electron-donating groups at the C2′ or C3′ position. However, the process for the formation of these compounds is less effective^11^. Nyangulu developed a novel ABA analog to inhibit the Michael addition of hydroxylated ABA *in vivo*. This compound, 2′,3′-benzoabscisic acid (PhABA, **2**), exhibited higher ABA-like activities against seed germination inhibition, stomatal movements and other bioassays than did ABA^12^. Thus, 2′,3′-PhABA is available and practical ABA analog due to its ease of preparation, but it is still sensitive to light isomerization.

In recent decades, great progress has been made in the study of ABA signal transduction^13^,^14^,^15^. Generally, ABA detection and signaling occurs by recruiting ABA receptors and protein phosphatases 2C (PP2Cs). PYL belongs to the START super-family, a large and evolutionarily ancient family of hydrophobic ligand-binding proteins that possess a conserved ‘helix-grip’ fold that contains a central α-helix surrounded by a 7-stranded anti-parallel β-sheet to form a central hydrophobic ligand–binding pocket^16^. There are 14 PYL ABA receptors in *A. thaliana*, including dimeric receptors (PYR1 and PYL1-3) and monomeric receptors (PYL4-13)^17^. The binding of ABA to the ligand-binding pocket of receptors leads to the transformation of the receptor conformation, involving the closure of a gate loop and latch loop^18^. This conformational transformation creates a surface for interaction with the active site of PP2Cs, including HAB1, HAB2, ABI1 and ABI2. The formation of a ternary ABA-PYLs-PP2C complex relieves PP2C-mediated inhibition of downstream SnRK2 kinases^19^,^20^,^21^. SnRK2 kinases, activated upon autophosphorylation, phosphorylate and activate the transcription factors that induce the expression of ABA-responsive genes^20^. Once activated, SnRK2s phosphorylate downstream targets including basic leucine zipper (bZIP)-type transcription factors and slow sustained (S-type) anion channels^22^. A series of ABA responses can subsequently be triggered. Previous studies have indicated that both dimeric and monomeric receptors are involved in plant ABA responses^23^,^24^. However, the physiological importance of ABA-independent PP2C inhibition by monomeric receptors remains unresolved. It has additionally been demonstrated that the selective chemical activation of dimeric receptors elicits a nearly complete ABA response, which points to dimeric receptors as key factors in ABA signaling^18^.

As part of continuous efforts to develop novel ABA analogs with high stability and high activity, we designed and synthesized 2,3-cyclopanated ABA analogs with high ABA-like activity and much higher photostability than ABA^25^,^26^. More recently, we also synthesized 2′,3′-PhABA **2** analogs^27^ and abscisic acid esters^28^. In particular, we synthesized *iso*-ABA **3**, an isomer of ABA that differs at the methyl position and exhibited nearly equivalent activity to ABA^29^. This suggests that the methyl position is not crucial for ABA-like activities, in agreement with the conclusion drawn by Wilmer^30^. Here, we described the design and functional characterization of 2′,3′-benzo-*iso*-ABA (*iso*-PhABA **4**) and demonstrate that it acts as an excellent selective ABA-receptor agonist (Fig. 1).

## Results

### Synthesis

**Figure 2:**
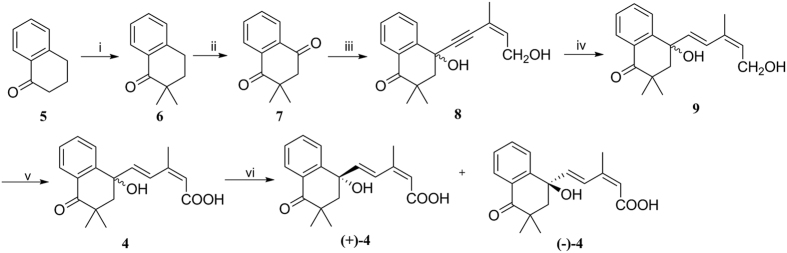
Synthesis of *iso-*PhABA 4. Reagents and conditions: i. MeI, NaH, THF; ii. Co(acac)_2_, *t-*BuOOH, acetone; iii. *n*-BuLi, (*Z*)-3-methylpent-2-en-4-yn-1-ol, THF, −78 °C; iv. Red-Al, THF, 0 °C; v. (**A**) DMP, DCM, r.t.; (**B**) NaClO_2_, 2-methyl-2-butene, NaH_2_PO_4_, *t*-BuOH:H_2_O = 3:1; vi. Chiral HPLC.

Based on the high ABA-like activities of PhABA **2** and *iso-*ABA **3**, coupling to acquire the antioxidation ABA analog *iso*-PhABA **4** was carried out using commercially available 1-tetralone **5** (Fig. 2). The germinal methyl groups were introduced adjacent to the carbonyl carbonby the treatment of **5** with methyl iodide in the presence of sodium hydride to give the dimethyl tetralone **6**. Dione **7** was obtained according to a reported method^31^. Alcohol **8** was synthesized by the highly efficient regioselective nucleophilic addition of alkynyl lithium to dione **7** based on steric effects^32^; **7** was formed *in situ* using (*Z*)-3-methylpent-2-en-4-yn-1-ol and *n*-butyl lithium. The selective reduction of the triple bond of **8** gave alcohol **9**. Oxidizing the racemic alcohol **9** to the corresponding acid **4** required a two-step procedure: Dess-Martin oxidation followed by a Lindgren oxidation^27^. The resolution of the racemic acid **4** using chirally preparative HPLC provided enantio pure (+)- and (−)-isomers of *iso-*PhABA **4**.

### Biological activity

As shown in Table 1, *iso-*PhABA **4**, especially (+)-*iso-*PhABA **4**, exhibited excellent ABA-like activities on the inhibitory effects on the seed germination of lettuce and *A. thaliana*, wheat embryo germination and rice seedling elongation. In these four assessments, (+)-*iso*-PhABA showed much higher activities than (+)-ABA, and the values of IC_50_ were lower than for (+)-ABA by a factor or at least two. In particular, for inhibiting lettuce seed germination, (+)-*iso*-PhABA displayed greater than 10-fold higher activity compared to (+)-ABA; the IC_50_ values were 0.15 μM and 1.63 μM, respectively. *In vivo*, ABA is inactivated by cytochrome P450–mediated hydroxylation by CYP707A enzymes^17^. The high activities of (+)-*iso-*PhABA might, therefore, be a consequence of decreased metabolic inactivation by CYP707A enzymes^33^. According to the very weak activities of (−)-ABA, it is reasonable that (−)-*iso*-PhABA presented much lower activities than its stereoisomer, but has considerable activity, especially for inhibiting *A. thaliana* seed germination (0.73 μM IC_50_). The affinity analysis showed that *iso*-PhABA had greater affinity than ABA (K_d_ values of (+)-*iso*-PhABA, (+)-*iso*-PhABA and (+)-ABA were 35.67, 13.46 and 62.63, respectively). This indicates that the interaction of *iso*-PhABA and ABA with receptors may differ. In our previous work, the biological activity and SAR of PhABA analogs were studied, PhABA **2** showed best activity. However, *iso*-PhABA **4** displayed better activity than PhABA **2**, which implied the side chain position changed of PhABA had significant effected.

### PYL10 exhibits high inhibition of PP2Cs in the presence of (+)/(−)*-iso-*PhABA 4

**Figure 3:**
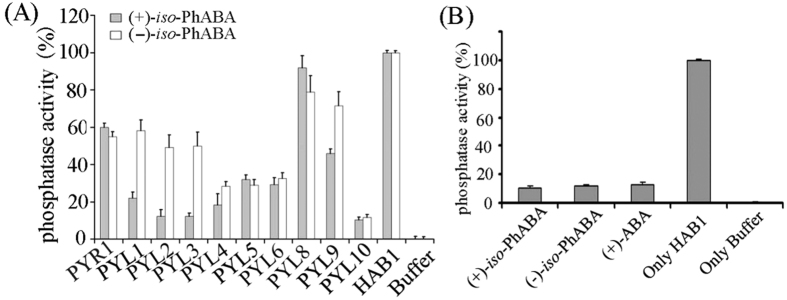
Subclass of PYLs Inhibits PP2Cs in the presence of (+)/(−)-*iso*-PhABA. (**A**) (+)/(−)-*iso*-PhABA mediated the inhibition of HAB1 phosphatase activity by PYLs. The concentration for each PYL protein was 5 μM and was 3.0 μM for HAB1 and 10 μM for (+)/(−)-*iso*-PhABA. All experiments were repeated three times (n = 3), and error bars represent the s.d. The conditions measuring the phosphatase activity of HAB1 were the same throughout, unless otherwise noted. The relative phosphatase activity of each reaction was normalized to that of the reaction with phosphopeptide substrate and HAB1 (100%). (**B**) PYL10 inhibits HAB1 phosphatase more strongly in the presence of (+)/(−)-*iso*-PhABA than (+)-ABA.

To further functionalize *iso*-PhABA, we examined the inhibitory effects of some PYLs on the phosphatase activity of HAB1 in the presence of (+)/(−)-*iso*-PhABA. The phosphatase activity was measured using the Ser/Thr phosphatase assay system. As shown in Fig. 3A, the tested PYLs could inhibit PP2Cphosphatase activity in the presence of (+)/(−)-*iso*-PhABA at moderate to high levels: the highest inhibition was presented by PYL 10, even slightly higher than for(+)-ABA (Fig. 3B). In binding to PYL1, PYL2, and PYL3, (+)-*iso*-PhABA showed much higher activity than (−)-*iso*-PhABA. In binding to PYR1, PYL5, PYL6 and PYL10, the two isomers showed nearly the same inhibitory activities. However, when binding to PYL9, (−)-*iso*-PhABA was more active than (+)-*iso*-PhABA. The results indicated that (+)/(−)-*iso*-PhABA might be selective agonists for ABA PYL receptors. Meanwhile, (−)-*iso*-PhABA showed considerable activity for PYLs receptors, differing greatly from (−)-ABA and showing little activity for *cis*-dimeric PYLs^34^. The results also suggested a different mechanism of action for *iso*-PhABA and ABA.

### Overall structures of (+)/(−)-*iso*-PhABA-bound PYL10

**Figure 4:**
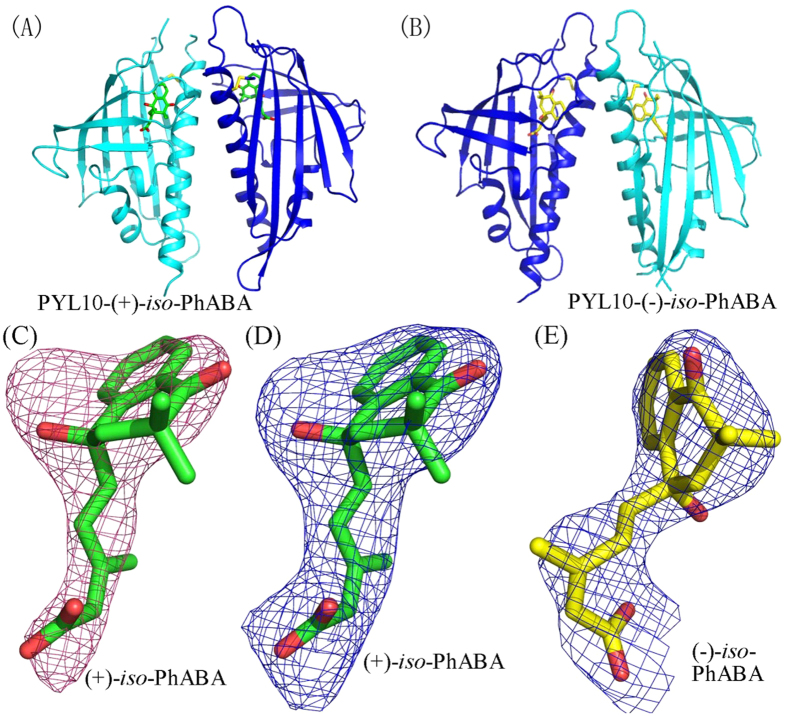
Overall structure of PYL10-(+)/(−)-*iso*-PhABA. Cartoons of PYL10-(+)-*iso*-PhABA (**A**) and PYL10-(−)-*iso*-PhABA (**B**). (**C**) *F*_*o*_−*F*_*c*_ differential electron density map of bound (+)-*iso*-PhABA at 2.5 σ. *2F*_*o*_−*F*_*c*_ electron density map of bound (+)-*iso*-PhABA (D) and (−)-*iso*-PhABA (E) at 1.0 σ. The (+)/(−)-*iso*-PhABA is represented by a stick model ((+)-*iso*-PhABA, green; (−)-*iso*-PhABA, yellow).

The isomers of (+)/(−)-*iso*-PhABA-bound PYL10 were crystallized in the P3_1_ space group (Fig. 4A and B, respectively). Just as apo-PYL10 and ABA-bound PYL10, the complex structures of (+)/(−)-*iso*-PhABA-PYL10 adopt a helix-grip fold composed of seven anti-parallel β-strands and two α-helices. The *iso*-PhABA isomer positions were found by the clear *F*_*o*_*-F*_*c*_ difference in electron density (Fig. 4C) and 2*F*_*o*_*-F*_*c*_ electron density (Fig. 4D and E) and further confirmed by the low thermal factors. The two isomers in the asymmetric unit of the PYL10-(+)/(−)-*iso*-PhABA complex were similar, with a root-mean-square deviation (RMSD) of 0.7 Å. The protomer structures were similar to the previously reported PYL10-(+)-ABA structure^35^, with a RMSD of 0.7 Å for 153 equivalent Cα atoms.

**Figure 5:**
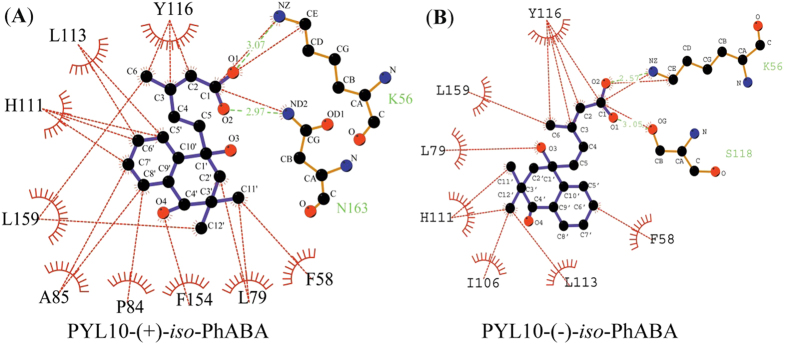
Detailed interactions between PYL10 and (+)-*iso*-PhABA (**A**) or (−)-*iso*-PhABA (**B**) *via* hydrogen bonds (green) and *van der Waals* interactions (red).

As shown in Fig. 5, the ligands (*iso*-PhABA) were arranged in the PYLs ligand-binding pocket through hydrogen bonds and *van der Waals* interactions. The carboxylic group of the ligands formed a hydrogen bond with the side chain amine group of K56 in PYL10. The isoprene moiety and the cyclohexene ring formed several hydrophobic interactions and hydrogen bonds with the side chains of F58, L79, P84, A85, H111, L113, Y116, I106, F154, L159, N163 and S118. Most of these residues were highly conserved in all 14 PYLs proteins (Fig. 5).

**Figure 6:**
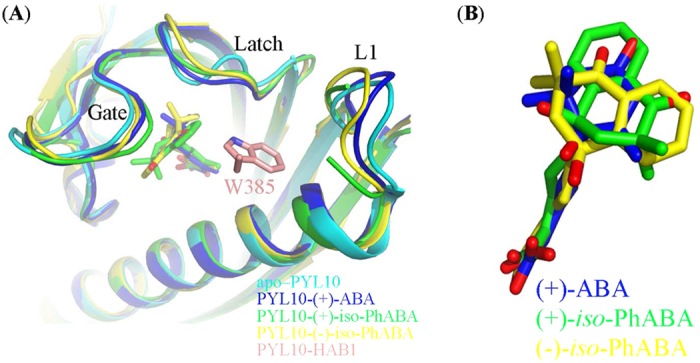
(**A**) Structural superimposition of apo–PYL10 (cyan, pdb ID: 3UQH), PYL10-(+)-ABA (blue, pdb ID: 3R6P), PYL10-(+)-*iso*-PhABA (green), PYL10-(−)-*iso*-PhABA (yellow) and PYL10-HAB1 (brown, pdb ID: 3RT0). (**B**) The (+)/(−)-*iso*-PhABA coordinated the same position in the pocket as the (+)-ABA molecule.

When apo-PYL10, ABA-bound PYL10 and (+)/(−)-*iso*-PhABA bound PYL10 were superimposed, the overall PYL10 structures were similar (Fig. 6A). Moreover, (+)/(−)-*iso*-PhABA was more coordinated in the same binding pocket than the (+)-ABA molecule (Fig. 6B). However, the gate and L1 loops (Fig. 6A) differed greatly. For example, upon (+)-*iso*-PhABA binding, the backbone and side chains of the gate loop residues in PYL10 moved approximately 1.9 Å towards the ligand. This movement was 0.8 Å further than for (+)-ABA. The gate movement further locked the ligand *iso*-PhABA tightly into the binding pocket. Moreover, the approximation of the L1 loop enhanced the binding of W385 in HAB1 (Fig. 6A).

### Hydrophobic interaction affects the selectivity of PYL10 for (+)/(−)-*iso*-PhABA

The phosphatase assay results showed that most PYLs were more strongly inhibited in the presence of (+)-*iso*-PhABAthan (−)-*iso*-PhABA; PYL10 had the strongest inhibition. Here, structures of PYL10-(+)/(−)-*iso*-PhABA were superimposed to illustrate the stereospecificity of PYL10 for both ABA enantiomers.

**Figure 7:**
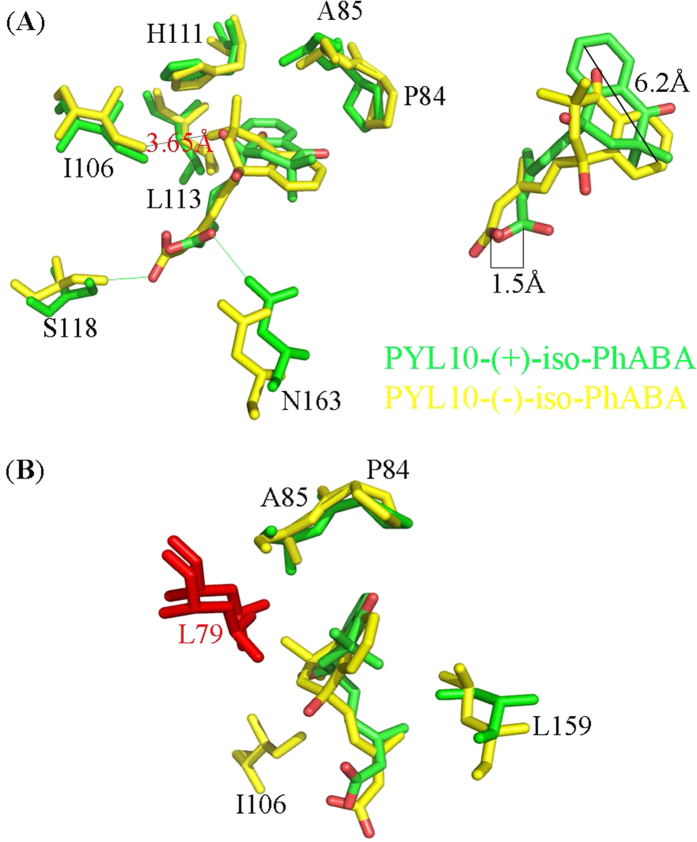
Identification of the molecular determinant of PYL10 for the selection of (+)/(−)-*iso*-PhABA. (**A**) Superposition of the binding pocket and the two (+)/(−)-*iso*-PhABAs (right panel). There were partial rotations and shifts between the rings and the carboxyl terminus in both *iso*-PhABAs. The (+)-*iso*-PhABA and its interacting residues in PYL10 are green, and the (−)-*iso*-PhABA and its interacting residues in PYL10 are yellow. (**B**) The residues affect (+)/(−)-*iso*-PhABA binding to PYLs. L79 (red), located at the center of a network of *van der Waals* contacts composed of residues from gate loop, latch loop, and α3 in PYL10.

First, (−)-*iso*-PhABA imitated the ring and tail orientations of (+)-*iso*-PhABA (Fig. 7A). However, the relative orientation between the benzene ring and the 11′,12′-dimethyl groups was reversed (Fig. 5), altering the hydrophobic interactions. Upon the binding of (+)-*iso*-PhABA, the benzene ring of (+)-*iso*-PhABA was confined to a very tight space in PYL10, which was coordinated by P84, A85, H111 and L113. However, upon the binding of (−)-*iso*-PhABA, the distance between the benzene ring and P84 and A85 became too great for hydrophobic interaction because the 11′,12′-dimethyl groups occupied this area. Only the H111, L113 and I106 residues had hydrophobic interactions with the 11′,12′-dimethyl group (Fig. 5). In addition, the benzene ring of (−)-*iso*-PhABA moved 6.2 Å, exposing it to the solvent (Fig. 7A right panel) compared to (+)-*iso*-PhABA, for which only the F58 residue formed hydrophobic interaction with the benzene ring of (−)-*iso*-PhABA. Thus, (−)-*iso*-PhABA was not limited to a tight space in PYL10 as was (+)-*iso*-PhABA. Second, in the pocket of (−)-*iso*-PhABA/PYL10, the 11′,12′-dimethyl groups were coordinated by I106, H111, and L113, but the distance between I106 and the 11′,12′-dimethyl groups of (+)-*iso*-PhABA was too great for hydrophobic interaction. However, the F58, L79 and L159 residues participated in the hydrophobic network to fix the dimethyl group in the (+)-*iso*-PhABA binding pocket. Third, the carboxyl group of (+)-*iso*-PhABA moved 1.5 Å closer to the α3 helix (Fig. 7A right panel), forming a hydrogen bond interaction with its N163, while the carboxyl group of (−)-*iso*-PhABA had a hydrogen bond interaction with the S118 in the β6 sheet. Finally, the L1 loop moved 0.8 Å closer to (+)-*iso*-PhABA compared to the (−)-*iso*-PhABA binding pocket, indicating the enhanced binding of W385 in HAB and the more effective inhibition of the phosphatase activity of HAB1 (Fig. 7A).

Altogether, the (+)-*iso*-PhABA made more hydrophobic interactions with the residues of the binding pocket in PYL10, and most of these residues were highly conserved in all 14 PYLs proteins. Thus, most PYLs bound more tightly to (+)-*iso*-PhABA than (−)-*iso*-PhABA.

### Identification of the molecular determinant for the preference of (+)/(−)-*iso*-PhABA to PYLs

To identify the factors that contribute to the (+)/(−)-*iso*-PhABA selective preference for various PYLs, we thoroughly examined the available structures. Generally, binding to and the inhibition of PP2Cs required the closure of the gate loop. We analyzed the sequence alignments of PYLs, particularly the residues forming contacts between the gate loop with the ligand. A good candidate was PYL10 residue L79 because a valine residue occupies this position in all other PYLs. L79 forms a direct hydrophobic interaction with the 11′,12′-dimethyl groups of (+)-*iso*-PhABA and the hydroxyl group of (−)-*iso*-PhABA (Fig. 5). In addition, the side chain of L79 in PYL10 made *van der Waals* contact with P84 and A85 in the gate loop. L159 in the α3 helix added to this network of interaction; the network formed by hydrophobic residues may effectively anchor the gate loop in a closed conformation (Fig. 7B). In other PYLs, the valine corresponding to residue L79 in PYL10 was too small to form hydrophobic interactions with the ligand and thus could not bind (+)/(−)-*iso*-PhABA strongly. Valine in other PYLs may not contact other hydrophobic residues to form a network that closes the gate loop. This was also found for the L159 of PYL10, as the corresponding residue in PYR1 and PYLs 1–3 is valine, and PYLs 4–12 contain either leucine or isoleucine. These side chains may have weaker interactions with the surrounding hydrophobic residues to close the gate loop. In the (+)-*iso*-PhABA binding pocket, the F154 in other molecules was important for locating small molecules, while the corresponding residue in other PYLs was either leucine, serine, methionine, all of which are too small to interact with (+)-*iso*-PhABA. However, in the (−)-*iso*-PhABA binding pocket, I106 confined the 11′,12′-dimethyl groups of the small molecule to a tight space that was too small for bulky side chains such as the phenylalanine residue in other PYLs.

Generally, the structural analyses support the notion that the hydrophobic residues at the entrance to the ligand-binding pocket form a network to close the gate loop, and the strong steric constraints in the binding pocket also affect the selectivity of (+)/(−)-*iso*-PhABA in PYLs. Thus, (+)-*iso*-PhABA and (−)-*iso*-PhABA are good selective agonists for PYLs.

In summary, we designed and functionalized the novel ABA analog 2′,3′-benzo-*iso*-ABA (*iso*-PhABA **4**). Notably, (+)-*iso*-PhABA showed much higher ABA-like activity than ABA itself. In the presence of (+)-*iso*-PhABA, PYLs showed moderate to high inhibitory activity against PP2Cs, suggesting that (+)-*iso*-PhABA is a selective ABA receptor agonist. The complex crystal structure of *iso*-PhABA with PYL10 revealed that (+)-*iso*-PhABA is more coordinated in the same binding pocket than (+)-ABA. Together with its convenient preparation, these findings indicated that (+)-*iso*-PhABA might be a potential ABA receptors agonist.

## Methods

### Synthesis

#### Materials and measurements

^1^H and ^13^C NMR spectra were recorded on a Bruker Avance DPX300 using tetramethylsilane as an internal standard. All NMR spectra were obtained using CDCl_3_ as the solvent unless otherwise noted. GC-MS was carried out on a 6890N GC-Agilent 5973N mass spectrometer. HPLC was carried out on an US Agilent 1100 instrument. Mass spectra were obtained with a VG-ZAB-HS mass spectrometer. Mass spectra data are reported in mass-to-charge units (*m*/*z*). High-resolution mass spectra (HRMS) were recorded in ESI mode using a Bruker Apex IV FTMS. Optical rotation was obtained using a Perkin Elmer 241MC polarimeter. Commercially available compounds were used in this work without further purification. Tetrahydrofuran (THF) and benzene were dried by distillation from sodium and benzophenone. CH_2_Cl_2_ (DCM) was dried by distillation with CaH_2_. Unless otherwise indicated, all reactions were conducted under dry nitrogen. Other instrumentation included a SANYO-autoclave, intelligent artificial climate chamber (Ningbo SAIFE Instruments Co. Ltd, P9X-250B) and a Super Clear Workbench (Donglian Electronic & Technology Development Co. Ltd, DL-CJ-1ND). MS (100 mL): 0.44 g (MS519), 3.0 g (sucrose) and 0.9 g (Agar) dissolved 100 mL of distilled water. *Arabidopsis thaliana* seeds were gifts from Prof. Xuechen Wang at the State Key Laboratory of Plant Physiology and Biochemistry.

### Protein expression and purification

PYR1 and PYL1 to PYL13 were sub-cloned from the *A. thaliana* DNA library using a standard PCR-based protocol. The fragments were inserted into the pET-28a vector or the pGEX-4T-2 vector. The thrombin recognition site was replaced by a TEV recognition site. The sequences of the insert were verified by DNA sequencing and transformed into *Escherichia coli* strain BL21 (DE3) for protein expression. Transformed cells were cultured at 37 °C in LB medium containing 50 μg/mL kanamycin or ampicillin. When the culture density reached an OD_600_ of 0.8–1.0, induction with 0.1 mM IPTG was performed, and cell growth continued for an additional 12 h at 18 °C. Cells were harvested by centrifugation at 3,000 g for 15 min, resuspended in lysis buffer (20 mM Tris-HCl pH 8.0, 200 mM NaCl, 2 mM DTT) and lysed by sonication. The lysate was centrifuged at 47,000 g for 20 min, and the supernatant was filtered through a 0.45 μM filter membrane to remove cell debris and other impurities. The filtrate was applied to a Profinity^TM^ IMAC Ni-Charged Resin column (Bio-Rad) and further purified using size exclusion chromatography (Superdex 200 HR10/300 GL, GE Healthcare).

PYL10 (residues 25–183) was inserted into the pET-28a vector. The expression and purification were the same as the full-length PYLs. To excise the 6× His tag, a small amount of 6× His tagged TEV protease was added and incubated on ice overnight. The digestion was added to a Profinity^TM^ IMAC Ni-Charged Resin column to remove TEV protease. The flow-through underwent further purification using anion exchange chromatography (Q Sepharose^TM^ High Performance, GE Healthcare) and size exclusion chromatography.

HAB1 (residues 169–511) was inserted into the pGEX-4T-2 vector. The thrombin recognition site was also replaced by a TEV recognition site, and the sequence of the insert was verified by DNA sequencing. The expression and purification were the same as those for the PYLs. The supernatant after filtration through a 0.45 μM filter membrane was applied to a Glutathione Sepharose 4 FF Resin column (GE Healthcare). This column was washed with twenty-fold bed volumes of lysis buffer. To excise the GST tag, a small amount of 6× His-tagged TEV protease was added to this column and incubated on ice overnight. The digestion was added to a Profinity^TM^ IMAC Ni-Charged Resin column to remove the TEV protease. The flow-through underwent further purification using size exclusion chromatography.

### Phosphatase activity assay

The phosphatase activity was measured using the serine-threonine phosphatase assay system (Promega V2460 kit). Each reaction was performed in 45 μL of reaction buffer (20 mM Hepes, pH 7.5, 150 mM NaCl and 5 mM MgCl_2_) containing 3 μM HAB1, 5 μM PYLs protein and 10 μM (+)/(−)-*iso-*PhABA, if required. After 30 min at room temperature, 5 μL of phosphorylated peptide substrate, supplied in the Promega kit, was added to the reaction system at 30 °C for 25 min. The reaction was terminated by the addition of 50 μL of molybdate dye/additive mixture, and the absorbance at 620 nm was measured 30 min later. The OD_620_ value of the reaction without HAB1 was set as the baseline, while the phosphatase activity of the reaction without PYLs was set as 100% for HAB1. Each reaction was repeated at least three times, and the error bars indicated standard deviations.

### Crystallization and data collection

To obtain the PYL10-(+)-*iso-*PhABA complex crystals, (+)-*iso-*PhABA was mixed with purified PYL10 at a 5:1 ratio and incubated on ice overnight. The mixture was concentrated to approximately 10 mg/mL. The crystallization screen conditions were obtained from commercial kits (Hampton Research and Emerald Biosystems) and some self-made products. Initial trails were performed using the sitting-drop vapor diffusion method at 20 °C and 4 °C. The crystallization-solution droplet comprised 1.0 μL of each reservoir solution and 1.0 μL of freshly purified target protein complex, which was equilibrated against 100 μL of reservoir solution. The complex crystals appeared in a well solution containing 10% *iso*-propanol, 0.1 M Na Hepes, pH 7.5, 20% PEG4000. The crystal was transferred into a well solution containing 20% glycol as a cryoprotectant solution and flash-cooled in liquid nitrogen before collecting data.

To obtain the PYL10-(−)-*iso-*PhABA complex crystals, the purified apo-PYL10 fragment was concentrated to approximately 10 mg/mL to screen crystals. The apo-PYL10 native crystal appeared in the reservoir solution containing 25% PEG3350, 0.1 M Tris-HCl, pH 8.5, 0.2 M (NH_4_)_2_SO_4_. The crystal was soaked into a solution comprising 1.0 μL of reservoir solution and 1.0 μL of 10 mM (−)-*iso-*PhABA mother liquor. After seven days, the crystal was transferred into a solution containing 20% glycol as a cryoprotectant solution and flash-cooled in liquid nitrogen before collecting data.

All crystal data were collected at the KEK beamline NE3A, SSRF beamline BL17U and BSRF beamline 1W2B. All data were integrated and scaled using the HKL2000 suite of programs^36^. Data collection statistics are summarized in Table 2.

### Structure determination

Using the apo-PYL10 structure (PDB code: 3UQH)^17^ as the search model, molecular replacement solutions for PYL10-(+)/(−)-*iso-*PhABA were found using MOLREP^37^. The model and ligands were built manually in the COOT program^38^ and the SKETCHER package in the CCP4 package^39^. We used the REFMAC5 program^40^ to refine the structure. Structure refinement statistics are shown in Table 2.

## Additional Information

**How to cite this article:** Han, X. *et al*. Design and Functional Characterization of a Novel Abscisic Acid Analog. *Sci. Rep.* **7**, 43863; doi: 10.1038/srep43863 (2017).

**Publisher's note:** Springer Nature remains neutral with regard to jurisdictional claims in published maps and institutional affiliations.

## Supplementary Material

Supporting Information

## Figures and Tables

**Figure 1:**
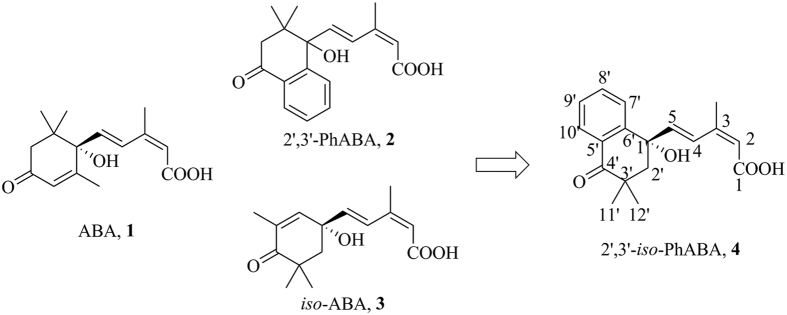
Design concept of *iso*-PhABA analogs.

**Table 1:** Bioactivities of *iso*-PhABA.

Bioassays	IC_50_ (μM)^a^				
	( ± )-*iso*-PhABA	(+)-*iso*-PhABA	(−)-*iso*-PhABA	( ± )-ABA	(+)-ABA
Lettuce seed germination	2.30	0.15	5.21	2.13	1.63
*Arabidopsis* seed germination	0.53	0.11	0.73	2.23	0.22
Wheat embryo germination	1.85	1.53	59.47	3.18	2.44
Rice seedlings elongation	1.17	0.55	6.62	1.34	1.01

^a^Concentration required to inhibit germination or seedlings elongation by 50%.

**Table 2:** Data collection and refinement statistics of PYL10 complexes*.

	PYL10-(+)-*iso-*PhABA	PYL10-(−)-*iso-*PhABA
**Data collection**		
Space group	*P*3_1_	*P*3_1_
**Cell dimensions**		
*a, b, c* (Å)	68.74, 68.74, 63.87	72.02, 72.02, 61.70
α, β, γ (˚)	90, 90, 120	90, 90, 120
Resolution (Å)	50.0–2.62 (2.67–2.62)^†^	50.0–2.85 (2.90–2.85)^†^
*R*merge (%)	8.1 (63.7)	10.0 (50.8)
*I*/σ*I*	35.4 (2.2)	32.9 (2.2)
Completeness (%)	98.3 (98.4)	95.4 (87.2)
Redundancy	7.8 (6.7)	5.5 (3.1)
**Refinement**		
Resolution (Å)	50.0–2.65 (2.72–2.65)	50.0–2.85 (2.92–2.85)
No. of reflections	9169 (662)	7586 (502)
*R*_work_/*R*_free_ (%)	24.6/27.5 (29.5/27.0)	22.6/26.4 (25.8/27.9)
**No**. **of atoms**		
Protein	1996	2025
Ligand	44	44
Water	18	6
***B*****-factors**		
Protein	81.85	84.78
Ligand/ion	77.07	88.8
Water	71.86	72.52
**rms deviations**		
Bond lengths (Å)	0.006	0.008
Bond angles (°)	0.995	1.291
Ramachandran Plot (%)^2^	84.7/15.3/0/0	86.5/13.5/0/0

*Three crystal experiments per structure.

Statistics for highest resolution shell.

^2^Residues in most favored, additionally allowed, generously allowed and disallowed regions of the Ramachandran plot.
