# Supplementary material for: Design and Functional Characterization of a Novel Abscisic Acid Analog
Source: Sci Rep. 2017 Mar 8;7:43863. doi: 10.1038/srep43863 (PMC5341028; doi:10.1038/srep43863)
Supplement: Supporting Information [file srep43863-s1.pdf]

Supporting Information for

Design and functional characterization of a novel Aabscisic Acid analog

Xiaoqiang Han,[1,3ξ] Lun Jiang,[2,4ξ] Chuanliang Che,[1] Chuan Wan,[1] Huizhe Lu,[1] Yumei Xiao,[1] Yanjun Xu,[1] Zhongzhou Chen,[2]* Zhaohai Qin[1]*

1. College of Science, China Agricultural University, Beijing 100193, China

2. College of Biological Sciences, China Agricultural University, Beijing 100193, China

3. College of Agricultural, Shihezi University, Shihezi 832000, China

4. Beijing Advanced Innovation Center for Food Nutrition and Human Health, China Agricultural University, Beijing100193, China

[ξ]These authors contributed same to the work.

* Corresponding author: qinzhaohai@263.net; chenzhongzhou@cau.edu.cn

*Tel.: +86-10-63732958. Fax: +86-10-63732958.

MATERIAL AND METHODS S3

1. Synthesis S3

1.1 Materials and measurements S3

1.2 Synthesis of *iso*-PhABA 4 .... S3

2. Bioassays S5

3. Receptor binding assay. S5

3.1 Protein expression and purification S5

3.2 Phosphatase activity assay S6

3.3 MST S7

3.4 Crystallization and data collection S7

3.5 Structure determination S8

NMR Spectra S10

Supplementary References S18

MATERIAL AND METHODS

1. Synthesis

1.1 Materials and measurements

The ^{1}H and ^{13}C NMR spectra were recorded on a Bruker Avance DPX300 with tetramethylsilane as an internal standard. All NMR spectra were obtained using $CDCl_3$ as the solvent unless otherwise noted. GC-Mass was carried out on a 6890N GC-Agilent 5973N. HPLC was carried US Agilent 1100. Mass spectra were obtained with a VG-ZAB-HS mass spectrometer. Mass spectra data are reported in mass to charge units (*m*/*z*). High-resolution mass spectra (HRMS) were recorded in ESI mode using Bruker Apex IV FTMS. Optical rotations were obtained from a Perkin Elmer 241MC polarimeter. Commercially available compounds were used in this work without further purification. The solvent tetrahydrofuran (THF) and benzene was dried by distillation from sodium and benzophenone. The CH_2Cl_2 (DCM) was dried by distillation CaH_2. Unless otherwise indicated, all reactions were conducted under dry nitrogen. SANYO-autoclave. Intelligent artificial climate chamber (NingBo SAIFE Instruments Co. Ltd, P9X-250B).Super Clear Workbanch (Donglian Electronic & Technology Development Co. Ltd, DL-CJ-1ND). MS (100mL): 0.44 g MS519, 3.0 g (sucrose) and 0.9g (Agar) dissolved 100 mL distilled water. (±)-ABA and *Arabidopsis thaliana* seeds were gifts from Prof. Xuechen Wang in State Key Laboratory of Plant Physiology and Biochemistry.

1.2 Synthesis of *iso*-PhABA 4

1.2.1 2,2-Dimethyl-3,4-dihydro-2H -naphthalen-1-one (**6**)

To a stirred suspension of NaH (11.6g, 334mmol, 70% in oil) in THF (25mL) in a 500mL round bottomed flask, was added, 1-tetralone **5** (10.0 g, 69mmol) dissolved in dry THF (20mL). After stirring the mixture for 10 min at r.t., methyl iodide (11.1mL, 178mmol) was added slowly. The mixture was then heated to 40℃ for 30min, and stirring continued at r.t. 3h. The reaction was quenched by addition of water (slowly and dropwise). The mixture then extracted with ethyl acetate (3×100mL), washed with water (2×100mL) and dried over anhydrous Na_2SO_4. Evaporation of the solvent yielded a brown oil. The residue was subjected to silica gel chromatography using PE and EtOAc (10:1) as eluant to afford 2,2-dimethyl-1-tetralone **6** (11.3g, 95%). ^{1}H NMR (300MHz, $CDCl_3$, 25°C) δ 8.03(d, 1H, *J*=7.5Hz), 7.45(t, 1H, *J*=7.5Hz), 7.28(t, 1H, *J*=7.5Hz), 7.21(d, 1H, *J*=7.5Hz), 2.97(t, 2H , *J*=6.5Hz), 1.97(t, 2H, *J*=6.5Hz), 1.20(s, 6H).

1.2.2 2,2-dimethyl-2,3-dihydronaphthalene-1,4-dione (**7**)

To a stirred solution of 2,2-dimethyl-1-tetralone **6** (8.0g, 46mmol), *t*-BuOOH (59.0g, 460mmol, 70%) and $Co(acac)_2$ (0.59g, 2.3mmol) in 100mL acetone at 28℃ for 15h. The mixture was then extracted with ethyl acetate (3×50mL), washed with water (2×50mL) and

dried over anhydrous Na_2SO_4. Evaporation of the solvent yielded a brown oil. The residue was subjected to silica gel chromatography using PE and EtOAc (6:1) as eluant to afford 2,2-dimethyl-2,3-dihydronaphthalene-1,4-dione **7** (5.62 g, 65%). ^{1}H NMR (300MHz, $CDCl_3$, 25°C) δ 8.09-8.05(m, 1H), 8.03-8,00 (m, 1H), 7.77-7.72(m, 2H), 2.94(s, 2H), 1.32(s, 6H). ^{13}C NMR (75MHz, $CDCl_3$, 25°C) δ 201.1, 196.1, 134.8, 134.3, 133.8, 133.6, 127.4, 126.0, 51.9, 45.4, 25.7, 25.6.

1.2.3 (*Z*)-(1'-hydroxy-3',3'-dimethyl-4'-oxo-tetrahydronaphthalene-one-yl)-3-methyl-pentyl-2-em-4-yn-1-ol (**8**)

To a stirred solution of (*Z*)-3-methylpent-2-en-4-yn-1-ol (0.5 g, 5.2mmol) in dry THF (25mL) was cooled to −78℃ under an atmosphere of argon. *n*-Butyl lithium (4.3mL, 10.4mmol, 2.4M in hexane) was then added slowly, *via* syringe. The mixture was allowed to stir at −78℃ for 45min, after which, 2,2-dimethyl-2,3-dihydronaphthalene-1,4-dione **7** (0.98g, 5.2mmol), dissolved in dry THF (5mL) was added. The mixture was stirred for a further 25min at −78℃ and then the ice bath was removed. The reaction mixture was stirred at r.t. for a further 3h. The reaction was quenched by addition of a saturated solution of NH_4Cl. The mixture was stirred for 10min and extracted with ethyl acetate (3×30mL), washed with water (2×20mL) and dried over anhydrous Na_2SO_4. Evaporation of the solvent yielded the desired alcohol as a brown oil. The residue was subjected to silica gel chromatography using PE and EtOAc (6:1) as eluant to afford (*Z*)-(1'-hydroxy-3',3'-dimethyl-4'-oxo-tetrahydronaphthalene-one-yl)-3-methyl-pentyl-2-em-4-yn-1-ol **8** (1.34 g, 91%). ^{1}H NMR(300MHz, $CDCl_3$, 25°C) δ 8.15(d, *J*=9.2Hz, 1H), 7.89(d, *J*=8.9Hz, 1H), 7.63(t, *J*=7.4Hz, 1H), 7.44(t, *J*=7.6Hz, 1H), 5.94-5.89(m, 1H), 4.28(d, *J*=6.6Hz, 2H), 2.97-2.88(m, 1H), 2.66-2.61(m, 1H), 1.91(d, *J*=1.1Hz, 3H), 1.76(s, 2H), 1.17 (s, 6H). ^{13}C NMR(75MHz, $CDCl_3$, 25°C) δ 197.1, 144.0, 136.5, 134.3, 130.0, 128.6, 127.0, 126.9, 120.0, 94.3, 85.9, 74.7, 61.25, 48.5, 41.4, 25.0, 23.1, 22.9.

1.2.4 (1*E*,3*Z*)-(1',4'-dihydroxy-3',3'-dimethyl-tetraloneyl)-3-methyl-pentyl-2-ene-4-yn-1-ol (**9**)

To a stirred solution of (*Z*)-(1'-hydroxy-3',3'-dimethyl-4'-oxo-tetrahydronaphthalene-one-yl)-3-methyl-pentyl-2-em-4- yn-1-ol **8** (0.95g, 3.3mmol) in dry THF was cooled to 0℃and Red-Al (2.8mL, 9.9mmol, 3.6M in toluene) added dropwise *via* syringe. The reaction mixture was stirred at r.t. for a further 3h. The reaction was quenched by slow addition of water (5mL) and extracted with diethyl ether (3×30mL). The organic phase was washed with water (2×20mL) and dried over anhydrous Na_2SO_4 and filtered, filtrate concentrated under reduced pressure. The residue was subjected to silica gel chromatography using PE and EtOAc (1:1) as eluant to afford (1*E*,3*Z*)-(1',4'-dihydroxy-3',3'-dimethyl-tetraloneyl)-3-methyl-pentyl-2-ene-4-yn-1-ol **9** (0.68g, 71%). ^{1}H NMR(300MHz, $CDCl_3$, 25°C) δ 7.55-7.52(m, 1H), 7.44-7.41(m, 1H), 7.33-7.26(m,

2H), 6.30(d, J=15.6Hz, 1H), 6.02(d, J=15.6Hz, 1H), 5.52(t, J=6.9Hz, 1H), 4.84(t, J=6.9Hz, 1H), 4.08(d, J=6.4Hz, 2H), 2.07-2.00(m, 2H), 1.83(s, 3H), 1.04(s, 3H), 0.99(s, 3H). ^{13}C NMR(75MHz, $CDCl_3$, 25°C) δ 140.5, 138.1, 135.6, 125.2, 128.1, 128.0, 127.6, 127.3, 127.2, 126.6, 78.8, 66.6, 58.0, 43.8, 39.0, 25.0, 22.5, 20.4.

1.2.5 (+)-*iso*-PhABA **4** and (-)-*iso*-PhABA **4**

To a stirred solution of (1*E*,3*Z*)-(1',4'-dihydroxy-3',3'-dimethyl-tetralone yl)-3-methyl-pentyl-2-ene-4-yn-1-ol **9** (0.5g, 1.74mmol), DMP (0.88g, 2.1mmol) in 20mL DCM at r.t. for 0.5h. After added 5mL aqueous $Na_2S_2O_3$ solution,10mL $CHCl_3$, aqueous $NaHCO_3$ solution, the resulting mixture, which was stirred for 10min, was extracted repeatedly with $CHCl_3$ (3×30mL). The collected organic extracts were washed with aqueous brine solution, dried, and concentrated under reduced pressure. The crude aldehyde was dissolved in 15mL solvent (t-BuOH:H_2O=3:1), stirred with 2-methyl-2-butene (2.38g, 34mmol), $NaClO_2$ (1.85g, 17.4mmol, 85%) and NaH_2PO_4•$2H_2O$ (1.08g, 6.96mmol) at rt for 10min. Extracted repeatedly with EtOAc (3×25mL). The collected organic extracts were washed with aqueous brine solution, dried, and concentrated under reduced pressure afford crude product. The residue was subjected to silica gel chromatography using PE, EtOAc and AcOH (1:1:0.1%) as eluant to afford *iso*-PhABA **4** (0.45 g, yield 86% over two steps). ^1H NMR(300MHz, $CDCl_3$, 25°C) δ 8.04-7.99(m, 1H), 7.78(d, J=16.0Hz, 1H), 7.58-7.52(m, 2H), 7.43-7.37(m, 1H), 6.42(d, J=16.0Hz, 1H), 5.73(s, 1H), 2.78(d, J=17.2Hz, 1H), 2.61(d, J=17.2Hz, 1H), 2.03(d, J=0.9Hz, 3H), 1.09(s, 3H), 1.06(s, 3H). ^{13}C NMR(75MHz, $CDCl_3$, 25°C) δ 197.5, 170.6, 151.8, 145.7, 139.3, 134.5, 130.9, 128.3, 128.2, 127.2, 126.7, 117.6, 78.3, 49.7, 41.1, 24.3, 23.4, 21.4. HRMS(m/z) $C_{15}H_{18}NaO_4$requires: 323.12593, found: 323.12538.

The enantiomers of *iso*-PhABA **4** were resolved by chiralHPLC (CHIRALCEL OZ-3 column, 4.6×250mm), Daicel Chemical Industries, Ltd., Hexane/EtOH/HAc=90/10/0.1 (V/V/V)) and had the following optical rotations:$[\alpha]_D^{25}$=+50.5 (*c* 1.0, $CHCl_3$) (retention time 6.916 min) and $[\alpha]_D^{25}$ = -63.6 (*c* 1.0, $CHCl_3$) (retention time 8.159 min) for(+)-*iso*-PhABA **4** and (−)-*iso*-PhABA **4**, respectively.

2. Bioassays.

The bioassays activities of the synthesized compounds were tested using our previously reported methods[1].

3. Receptor binding assay.

3.1 Protein expression and purification

PYR1 and PYL1 to PYL13 were subcloned from the *Arabidopsis thaliana* cDNA library using standard PCR-based protocol. The fragments were inserted into the pET-28a vector or pGEX-4T-2 vector, in which the thrombin recognition site was replaced by TEV recognition

site. The sequences of the insert were verified by DNA sequencing and transformed into *Escherichia coli* strain BL21 (DE3) for protein expression. Transformed cells were then cultured at 37 ℃ in LB medium containing 50μg/mL kanamycin or ampicillin. When the culture density reached an OD_{600} of 0.8-1.0, induction with 0.1mM IPTG was performed, and cell growth continued for an additional 12h at 18 ℃. Cells were harvested by centrifugation at 3000g for 15min, and then resuspended in lysis buffer (20mM Tris-HCl pH 8.0, 200mM NaCl, 2mM DTT) and lysed by sonication. The lysate was centrifuged at 47000g for 20min and the supernatant was filtrated by 0.45μM filter membrane to remove cell debris and other impurities, and then applied to Profinity™ IMAC Ni-Charged Resin column (Bio-Rad), then further purified by size exclusion chromatography (Superdex 200 HR10/300 GL, GE Healthcare).

PYL10 (residues 25-183) was inserted into the pET-28a vector, expression and purification were the same as the full length PYLs. To excise 6×His tag, a small amount of 6×His tagged TEV protease was added and incubated on ice overnight. The digestion production went through Profinity™ IMAC Ni-Charged Resin column to remove TEV protease. The flow through underwent a further purification step of anion exchange chromatography (Q Sepharose™ High Performance, GE Healthcare) and size exclusion chromatography.

HAB1 (residues 169-511) was inserted into the pGEX-4T-2 vector in which the thrombin recognition site was also replaced by TEV recognition site and the sequence of the insert was verified by DNA sequencing. The expression and purification were the same to those of PYLs. The supernatant after filtration by 0.45μM filter membrane was applied to Glutathione Sepharose 4 FF Resin column (GE Healthcare). Then this column was washed with twenty-fold bed volume lysis buffer. To excise GST tag, a small amount of 6×His tagged TEV protease was added into this column and incubated on ice overnight. The digestion production went through Profinity™ IMAC Ni-Charged Resin column to remove TEV protease. The flow through underwent a further purification step of size exclusion chromatography.

3.2 Phosphatase activity assay

The phosphatase activity was measured by the serine-threonine phosphatase assay system (Promega V2460 kit). Each reaction was performed in a 45 μL reaction buffer (20mMHepes pH 7.5, 150mM NaCl and 5mM $MgCl_2$) containing 3μM HAB1, 5μM PYLs protein and (+)/(-)-*iso*-PhABA of 10μM concentration if required. After 30min at room temperature, 5μL phosphorylated peptide substrate supplied with the Promega kit was added into the reaction system at 30 ℃ for 25min. And then the reaction was terminated by addition of 50μL molybdate dye/additive mixture, and the absorbance at 620nm was measured 30min later. The OD_{620} value of the reaction without HAB1 was set as baseline while the phosphatase activity

of the reaction without PYLs was set as 100% for HAB1. Each reaction was repeated at least three times and the error bars indicated standard deviations.

3.3 MST

The microscalethermophoresis (MST) method has been described in detailelsewhere[2]. The KD for binding of ligands and PYLs were measured using the Monolith NT.115 from Nanotemper Technologies. Proteins were fluorescently labeled according to the manufacturer's protocol. A solution of ligand was seriallydiluted from about 2 mM to 244 nM in the presence of 50 nM labeled PYLs.The samples were loaded into silica capillaries (Polymicro Technologies) after incubation at room temperature for 5 min. Measurements were performed at20℃ in 20m MTris buffer, pH 8.0, with 150 mM NaCl and0.05% Tween 20, by using 30% LED power and 20% IR-laser power. Data analyses were performed using Nanotemper Analysis software, v.1.2.101.

Table 1 The Kd values of ABA/*iso*-PhABA and PYLs

	PYL1	PYL2	PYL5	PYL10	PYR1
(+)-ABA	7.81	19.23	66.63	65.63	87.24
(+)-*iso*-PhABA	2.26	80.00	197.71	35.67	98.00
(-)-*iso*-PhABA	4.30	35.30	32.97	13.47	53.50

3.4 Crystallization and data collection

To get the PYL10-(+)-*iso*-PhABA complex crystals, (+)-*iso*-PhABA was mixed with purified PYL10 at 5:1 ratio and then incubated on ice overnight. The mixture was concentrated to about 10mg/mL.The crystallization screen conditions were from commercial kits (Hampton Research and Emerald Biosystems) and some self-made products. Initial trails were performed by sitting-drop vapor diffusion method at 20℃ and 4℃, respectively. Crystallization-solution droplet was comprised of 1.0μL each reservoir solution and 1.0μL freshly purified target protein complex, which was equilibrated against 100μL each reservoir solution. The complex crystals appeared in a well solution contained 10% *iso*-propanol, 0.1M Na Hepes pH 7.5, 20% PEG4000. The crystal was transferred into well solution containing 20% glycol as cryo-protectant solution and flash-cooled in liquid nitrogen before collecting data.

To get the PYL10-(-)-*iso*-PhABA complex crystals, purified apo-PYL10 fragment was concentrated to about 10mg/mL for screening crystals. Apo-PYL10 native crystal appeared in the reservoir solution contained 25% PEG3350, 0.1MTris-HCl pH 8.5, 0.2M $(NH_4)_2SO_4$. The crystal was soaked into solution which was comprised of 1.0μL reservoir solution and 1.0μL 10mM (-)-*iso*-PhABA mother liquor. After seven days, the crystal was transferred into

solution containing 20% glycol as cryo-protectant solution and flash-cooled in liquid nitrogen before collecting data.

All the crystal data were collected at KEK beamline NE3A, SSRF beamline BL17U and BSRF beamline 1W2B. All the data were integrated and scaled with the HKL2000 suite of programs[3]. Data collection statistics are summarized in Table 1.

Table 2. Data collection and refinement statistics of PYL10 complexes*

	PYL10-(+)-*iso*-PhABA	PYL10-(-)-*iso*-PhABA
**Data collection**		
Space group	$P3_1$	$P3_1$
Cell dimensions		
a, *b*, *c*, Å	68.74, 68.74, 63.87	72.02, 72.02, 61.70
α, β, γ, °	90, 90, 120	90, 90, 120
Resolution, Å	50.0-2.62 (2.67-2.62) †	50.0-2.85 (2.90-2.85) †
*R*merge, %	8.1 (63.7)	10.0 (50.8)
$I/\sigma I$	35.4 (2.2)	32.9 (2.2)
Completeness, %	98.3 (98.4)	95.4 (87.2)
Redundancy	7.8 (6.7)	5.5 (3.1)
**Refinement**		
Resolution, Å	50.0-2.65 (2.72-2.65)	50.0-2.85 (2.92-2.85)
No. of reflections	9169 (662)	7586 (502)
R_{work}/R_{free}, %	24.6/27.5 (29.5/27.0)	22.6/26.4 (25.8/27.9)
No. of atoms		
Protein	1996	2025
Ligand	44	44
Water	18	6
B-factors		
Protein	81.85	84.78
Ligand/ion	77.07	88.8
Water	71.86	72.52
rms deviations		
Bond lengths, Å	0.006	0.008
Bond angles, °	0.995	1.291
Ramachandran Plot, %[2]	84.7/15.3/0/0	86.5/13.5/0/0

* Three crystal experiments for each structure

[1] Statistics for highest resolution shell.

[2]Residues in most favored, additional allowed, generously allowed and disallowed regions of the Ramachandran plot.

3.5 Structure determination

Using apo-PYL10 structure (PDB code: 3UQH)[4] as the search model, molecular replacement solutions of the PYL10-(+)/(-)-*iso*-PhABA were found using MOLREP[5]. The model and ligands were built manually in the program COOT[6] and SKETCHER package in

the CCP4 package[7]. We used the program REFMAC5[8] to refine the structure. Structure refinement statistics are shown in Table 1.

NMR Spectra

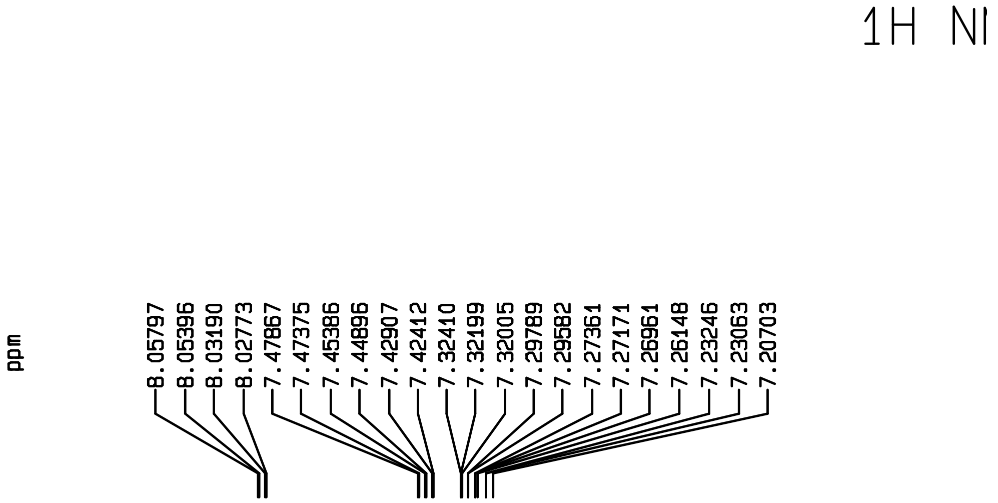

Figure 1.^{1}H NMR of compound **6**

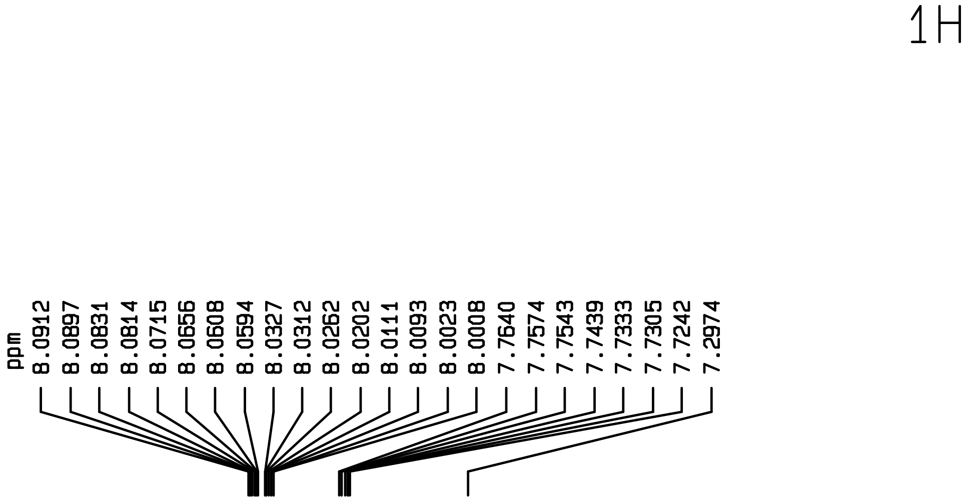

Figure 2.^{1}H NMR of compound **7**

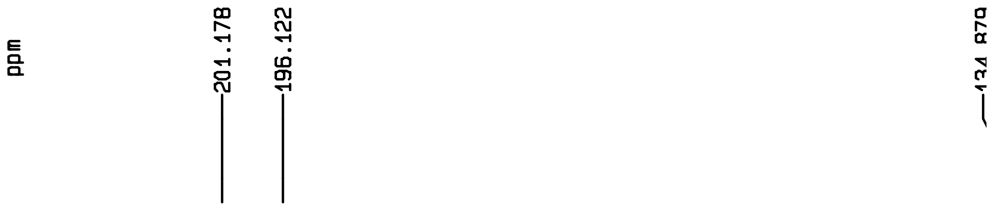

Figure 3.^{13}C NMR of compound **7**

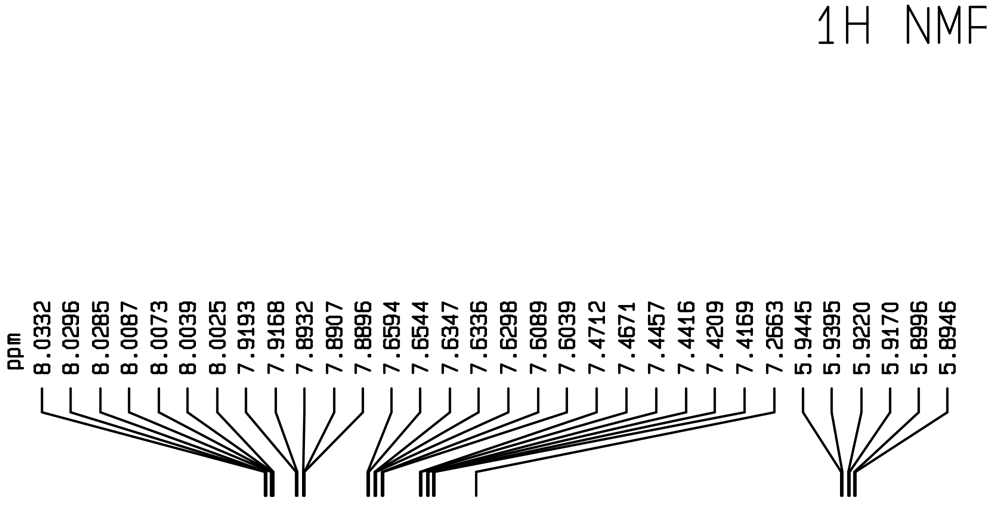

Figure 4.^{1}H NMR of compound **8**

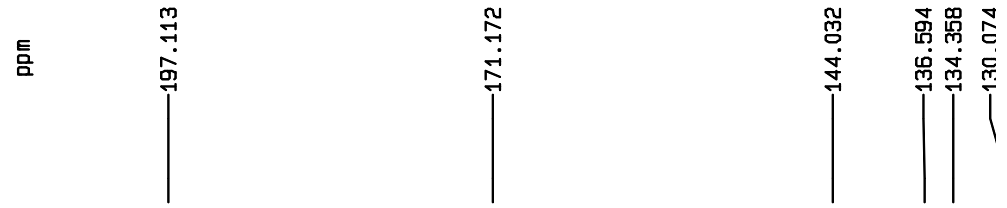

Figure 5.^{13}C NMR of compound **8**

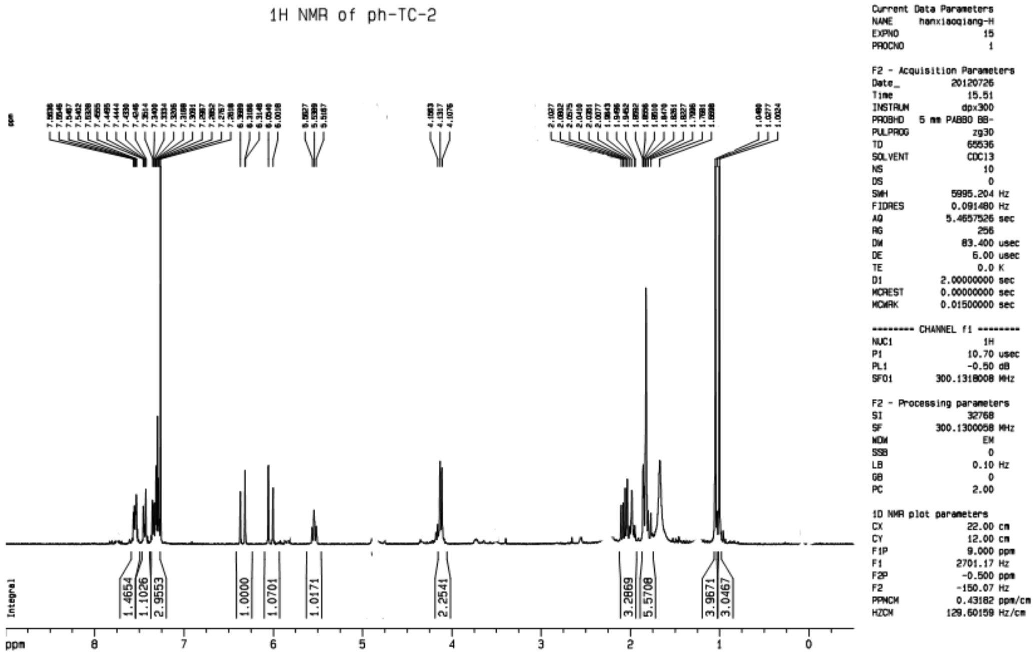

Figure 6.^{1}H NMR of compound **9**

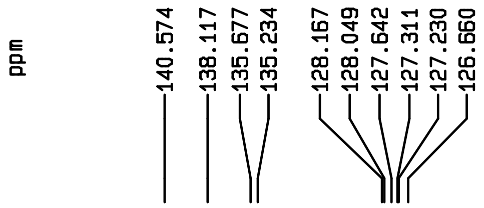

Figure 7.^{13}C NMR of compound **9**

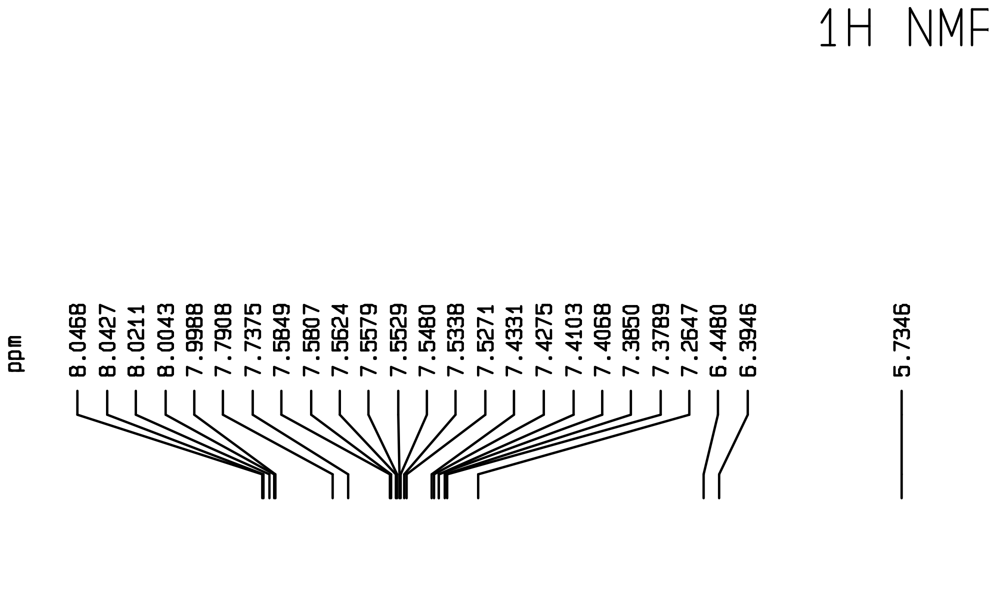

Figure 8.^{1}H NMR of *iso*-PhABA**4**

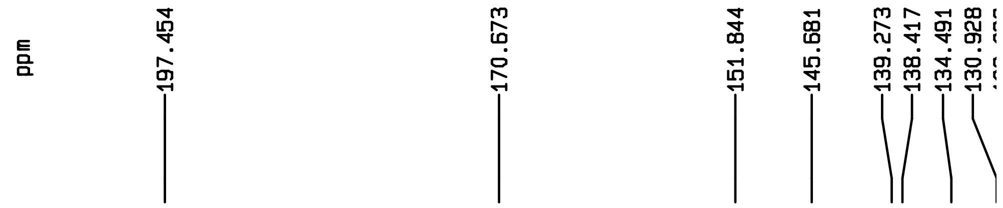

Figure 9.^{13}C NMR of *iso*-PhABA**4**

Peking University Mass Spectrometry Sample Analysis Report

Analysis Info

Analysis Name	12110569_20121120_000001.d	Acquisition Date	11/20/2012 3:42:33 PM
Sample	cau-1	Instrument	Bruker Apex IV FTMS
Comment	ESI Positive	Operator	Peking University

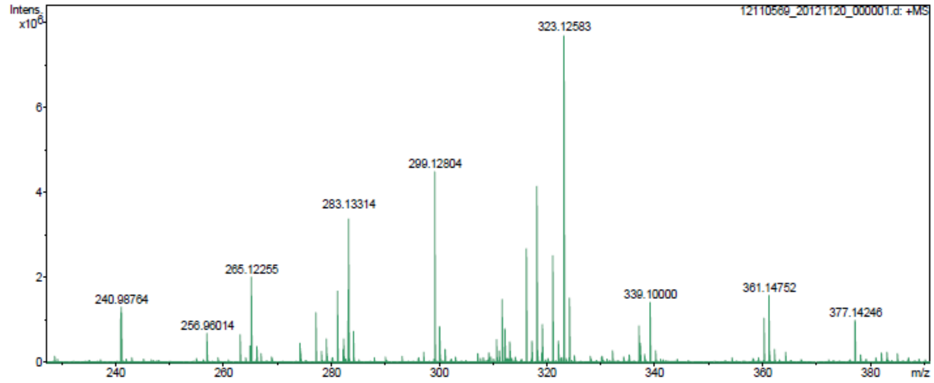

Meas. m/z	#	Formula	Score	m/z	err [mDa]	err [ppm]	mSigma	rdb	e¯ Conf	N-Rule
323.12583	1	C 18 H 20 Na O 4	100.00	323.12538	-0.5	-1.4	3.1	8.5	even	ok

Bruker Compass DataAnalysis 4.0 printed: 11/20/2012 3:44:42 PM Page 1 of 1

Figure 10. HRMS of *iso*-PhABA**4**

CHIRAL CHROMATOGRAPHY REPORT

(+)-*iso*-PhABA 4

Column	:	CHIRALCEL OZ-3
Column size	:	0.46 cm I.D. × 25 cm L
Injection	:	2 μL
Mobile phase	:	Hexane/EtOH/HAc=90/10/0.1（V/V/V）
Flow rate	:	0.9 mL/min
Wave length	:	UV 254 nm
Temperature	:	35℃
Sample solution	:	X mg/ml in mobile phase
Sample structure	:	Peak 1

< Chromatogram >

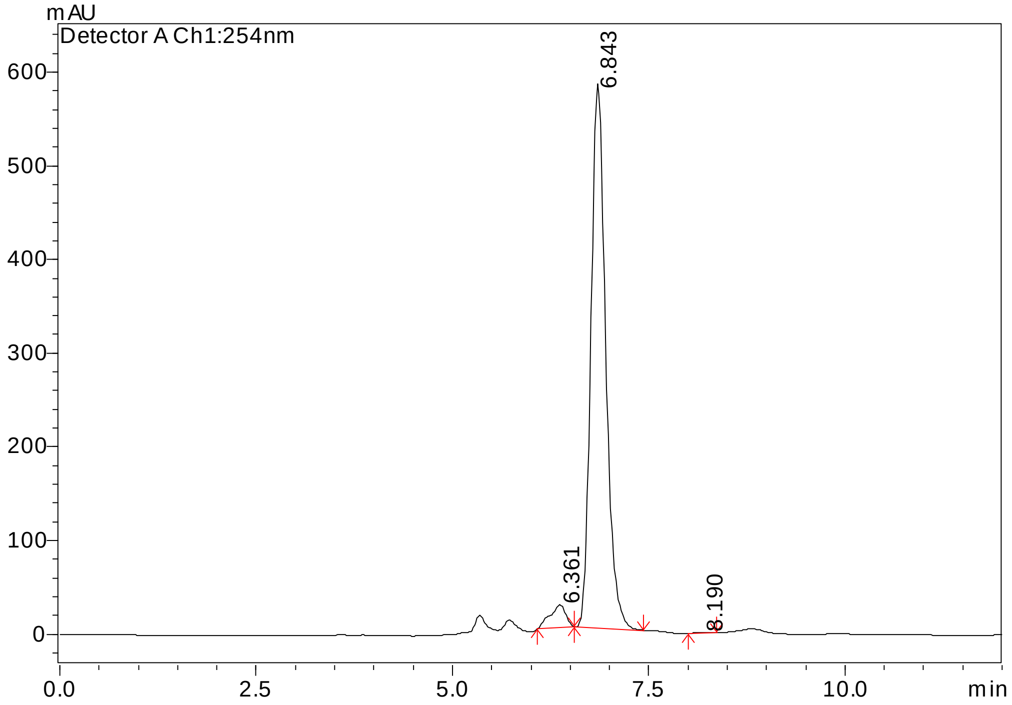

<Column Performance Report>

Peak No.	Time	Area	Area %	T Plate	Tailing	Resolution
1	6.361	345910	4.3217	3933.18	0.820	--
2	6.843	7645718	95.5236	5546.05	1.263	1.247
3	8.190	12382	0.1547	9115.85	0.940	3.792

Figure 11. HPLC of (+)-*iso*-PhABA**4**

CHIRAL CHROMATOGRAPHY REPORT

(-)-*iso*-PhABA 4

Column	:	CHIRALCEL OZ-3
Column size	:	0.46 cm I.D. ×25 cm L
Injection	:	2μL
Mobile phase	:	Hexane/EtOH/HAc=90/10/0.1（V/V/V）
Flow rate	:	0.9 mL/min
Wave length	:	UV 254 nm
Temperature	:	35℃
Sample solution	:	X mg/ml in mobile phase
Sample structure	:	Peak 2 收集液

< Chromatogram >

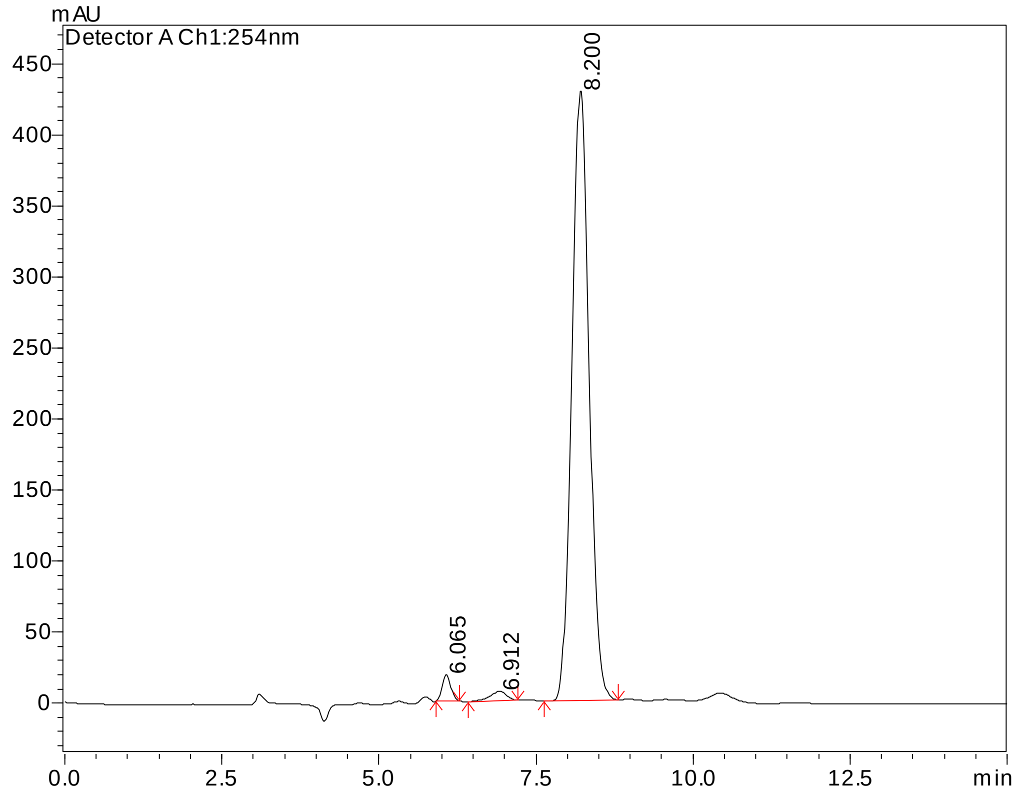

<Column Performance Report>

Peak No.	Time	Area	Area %	T Plate	Tailing	Resolution
1	6.065	178618	2.0849	7052.43	1.167	--
2	6.912	125233	1.4618	3075.80	0.837	2.150
3	8.200	8263430	96.4534	3871.68	1.099	2.513

Figure 12. HPLC of (-)-*iso*-PhABA**4**

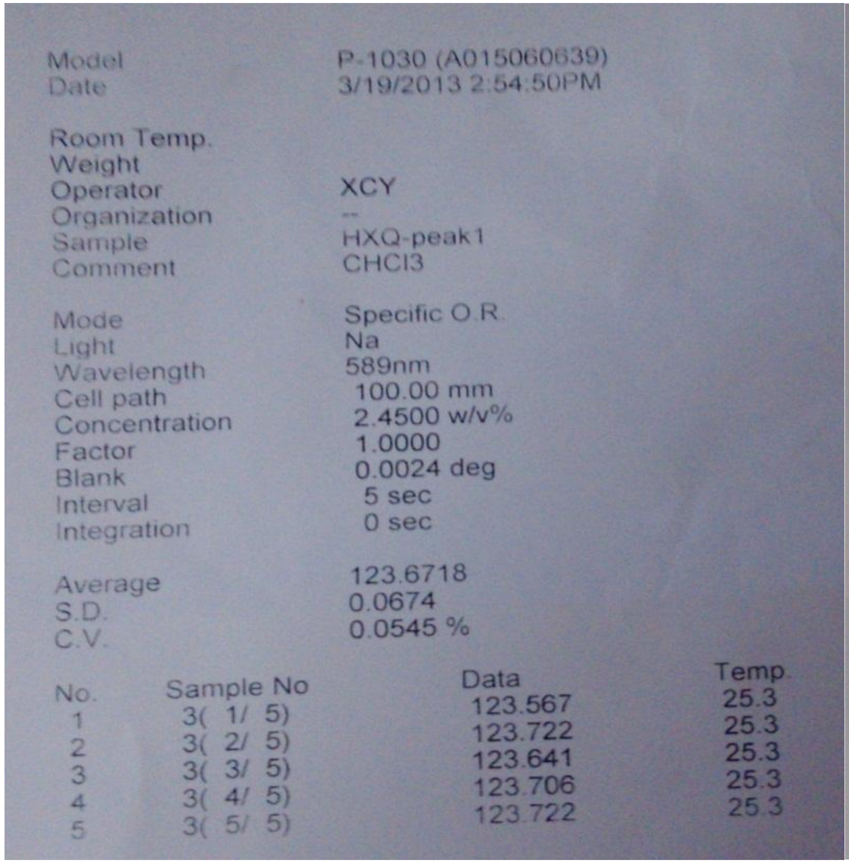

Model	P-1030 (A015060639)
Date	3/19/2013 2:54:50PM
Room Temp.	
Weight	
Operator	XCY
Organization	--
Sample	HXQ-peak1
Comment	CHCl3
Mode	Specific O.R.
Light	Na
Wavelength	589nm
Cell path	100.00 mm
Concentration	2.4500 w/v%
Factor	1.0000
Blank	0.0024 deg
Interval	5 sec
Integration	0 sec
Average	123.6718
S.D.	0.0674
C.V.	0.0545 %

No.	Sample No	Data	Temp.
1	3(1/ 5)	123.567	25.3
2	3(2/ 5)	123.722	25.3
3	3(3/ 5)	123.641	25.3
4	3(4/ 5)	123.706	25.3
5	3(5/ 5)	123.722	25.3

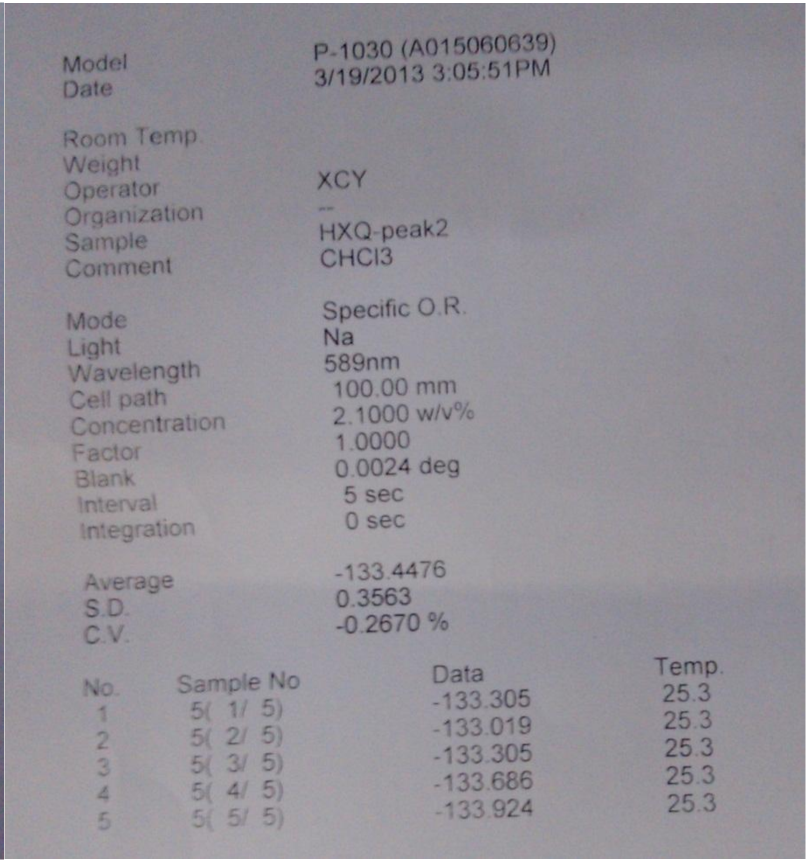

Model	P-1030 (A015060639)
Date	3/19/2013 3:05:51PM
Room Temp.	
Weight	
Operator	XCY
Organization	--
Sample	HXQ-peak2
Comment	CHCl3
Mode	Specific O.R.
Light	Na
Wavelength	589nm
Cell path	100.00 mm
Concentration	2.1000 w/v%
Factor	1.0000
Blank	0.0024 deg
Interval	5 sec
Integration	0 sec
Average	-133.4476
S.D.	0.3563
C.V.	-0.2670 %

No.	Sample No	Data	Temp.
1	5(1/ 5)	-133.305	25.3
2	5(2/ 5)	-133.019	25.3
3	5(3/ 5)	-133.305	25.3
4	5(4/ 5)	-133.686	25.3
5	5(5/ 5)	-133.924	25.3

Figure 13.Optical rotation of (+)-*iso*-PhABA**4**and (-)-*iso*-PhABA**4**

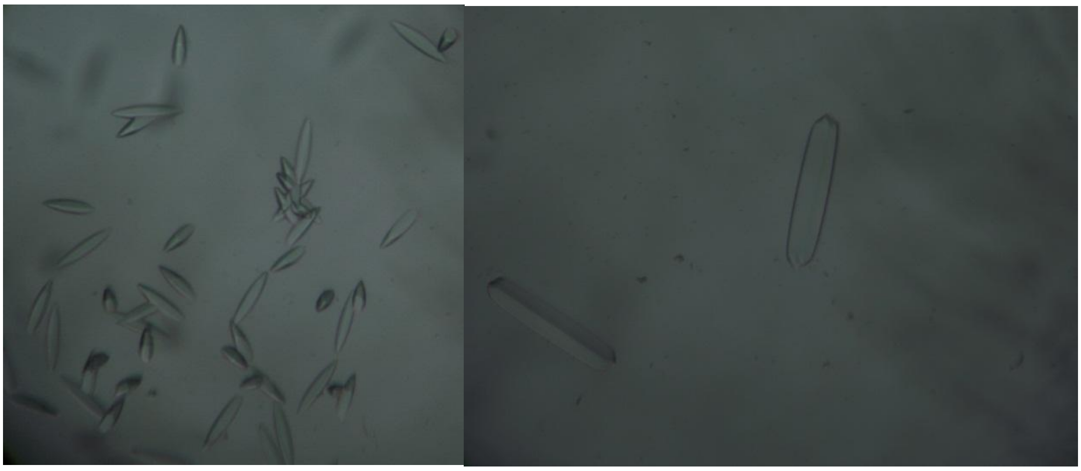

Figure 14.Complex structures of PYL10 with (+)/(-)-*iso*-PhABA**4**

Supplementary References

(1) Liu, W. J., Han, X. Q., Xiao, Y. M., Fan, J. L., Zhang ,Y. Z., Lu, H. H., Wang, M. A., Qin, Z. H. Synthesis, Photostability and Bioacivity of 2,3-CyclopropantedAbscisic Acid. *Phytochemistry* **96**, 72-80 (2013).

(2) Jerabek W. M., Wienken, C.J., Braun, D., Baaske, P., and Duhr, S. Molecular interaction studies using microscale thermophoresis. Assay Drug Dev. Technol. **9**, 342–353 (2011).

(3) Otwinowski, Z., Minor, W. Processing of X-ray diffraction data collected in oscillation mode. In: Charles, W., Carter, J., Sweet, R. M. eds. Methods in Enzymology. Academic Press, 307-326 (1997).

(4) Sun, D., Wang, H., Wu, M., Zhang, J., Wu, F., Tian, C. Crystal structures of the Arabidopsis thaliana abscisic acid receptor PYL10 and its complex with abscisic acid. *Biochem. Biophys. Res. Commun*. **418**, 122-127 (2012).

(5)Vagin, A.,Teplyakov, A. MOLREP: an Automated Program for Molecular Replacement. *J Appl. Crystallogr*. **30**, 1022-1025 (1997).

(6) Emsley, P., Cowtan, K. Coot: model-building tools for molecular graphics. *Acta Crystallogr D* **60**, 2126-2132 (2004).

(7)Winn, M. D., Ballard, C. C., Cowtan, K. D., Dodson, E. J., Emsley, P., Evans, P. R., Keegan, R. M., Krissinel, E. B., Leslie, A. G. W., McCor, A., McNicholas, S. J., Murshudov, G. N., Pannu, N. S., Potterton, E. A., Powell, H. R., Read, R. J., Vagin, A., Wilson, K. S. Overview of the CCP4 suite and current developments. *Acta Crystallogr D* **67**, 235-242 (2011).

(8)Murshudov, G. N.; Vagin, A. A.; Dodson, E. J. Refinement of Macromolecular Structures by the Maximum-Likelihood Method. *Acta Crystallogr D* **53**, 240-255 (1997).
